# Transparent Microelectrode Arrays Fabricated by Ion Beam Assisted Deposition for Neuronal Cell In Vitro Recordings

**DOI:** 10.3390/mi11050497

**Published:** 2020-05-14

**Authors:** Tomi Ryynänen, Ropafadzo Mzezewa, Ella Meriläinen, Tanja Hyvärinen, Jukka Lekkala, Susanna Narkilahti, Pasi Kallio

**Affiliations:** 1 Micro and Nanosystems Research Group, Faculty of Medicine and Health Technology, Tampere University, 33720 Tampere, Finland; ella.merilainen@tuni.fi (E.M.); jukka.lekkala@tuni.fi (J.L.); pasi.kallio@tuni.fi (P.K.); 2 Neuro Group, Faculty of Medicine and Health Technology, Tampere University, 33520 Tampere, Finland; ropafadzo.mzezewa@tuni.fi (R.M.); tanja.k.hyvarinen@helsinki.fi (T.H.); susanna.narkilahti@tuni.fi (S.N.)

**Keywords:** microelectrode array (MEA), ion beam assisted electron beam deposition (IBAD), indium tin oxide (ITO), titanium nitride (TiN), neurons, transparent

## Abstract

Microelectrode array (MEA) is a tool used for recording bioelectric signals from electrically active cells in vitro. In this paper, ion beam assisted electron beam deposition (IBAD) has been used for depositing indium tin oxide (ITO) and titanium nitride (TiN) thin films which are applied as transparent track and electrode materials in MEAs. In the first version, both tracks and electrodes were made of ITO to guarantee full transparency and thus optimal imaging capability. In the second version, very thin (20 nm) ITO electrodes were coated with a thin (40 nm) TiN layer to decrease the impedance of Ø30 µm electrodes to one third (1200 kΩ → 320 kΩ) while maintaining (partial) transparency. The third version was also composed of transparent ITO tracks, but the measurement properties were optimized by using thick (200 nm) opaque TiN electrodes. In addition to the impedance, the optical transmission and electric noise levels of all three versions were characterized and the functionality of the MEAs was successfully demonstrated using human pluripotent stem cell-derived neuronal cells. To understand more thoroughly the factors contributing to the impedance, MEAs with higher IBAD ITO thickness as well as commercial sputter-deposited and highly conductive ITO were fabricated for comparison. Even if the sheet-resistance of our IBAD ITO thin films is very high compared to the sputtered one, the impedances of the MEAs of each ITO grade were found to be practically equal (e.g., 300–370 kΩ for Ø30 µm electrodes with 40 nm TiN coating). This implies that the increased resistance of the tracks, either caused by lower thickness or lower conductivity, has hardly any contribution to the impedance of the MEA electrodes. The impedance is almost completely defined by the double-layer interface between the electrode top layer and the medium including cells.

## 1. Introduction

Microelectrode or multielectrode arrays (MEAs) are a common measurement platform in various in vitro studies where, for example, neuronal cells or cardiomyocytes are applied for drug screening, toxicity testing, cell model development or simply for increasing understanding of cell behavior [1,2,3,4]. The field potential [5] or impedimetric [6] measurements typically performed with MEA are usually complemented with fluorescence imaging or microscopic inspection during or after the MEA recordings. In such studies, the use of an inverted microscope is preferred as imaging from the top side is often impossible because of the cell culturing medium and its reservoir placed on top of the MEA. With the inverted microscope, there exists, however, another challenge. Typically, the tracks and especially the electrodes of the MEA are opaque and thus they prevent the full visibility of the cells from the bottom side—just where the electrical signal is originating and imaging the cells is usually of the greatest interest.

A common and unfortunately only a partial solution to the above problem is to make the tracks from transparent indium tin oxide (ITO) [7,8,9] material. However, ITO is rarely used as the electrode material [10,11], simply because of the relatively high impedance and noise level of the ITO electrodes. Thus, opaque low-impedance platinum black [12] or titanium nitride (TiN) [13] electrodes are usually used with ITO tracks. Recently both Ryynänen et al. [14] and Mierzejewski et al. [15] have proposed the use of a very thin TiN layer on the electrodes, which would benefit from TiN’s columnar structure and thus capability of decreasing impedance by increasing the effective surface area, while still maintaining the transparency, at least to some extent. Graphene [16,17], diamond [18] and conducting polymers [19,20] have also been demonstrated as potential candidates for transparent electrodes, but they have their own challenges especially related to the ease of fabrication and stability.

Sputter deposition is the standard method for depositing both ITO [21,22] and TiN [13] thin films. However, we have successfully shown that ion beam assisted electron beam deposition (IBAD) can also be used as a deposition method in fabrication of opaque TiN electrodes [23]. In this paper, IBAD TiN has been applied not only as an opaque layer, but also as a very thin transparent top layer for the ITO electrodes. In addition, a process for depositing ITO by IBAD was developed. Apart from showing that IBAD is a valid alternative for sputtering in depositing both ITO and TiN for MEAs, this paper also focuses on evaluating the performance of different combinations of ITO tracks, as well as ITO and TiN electrodes, both from imaging and impedance points of view. Biocompatibility of the MEAs was verified by performing cell experiments with human pluripotent stem cell-derived (hPSC) neuronal cells.

## 2. Materials and Methods

### 2.1. MEA Design and Fabrication

**Figure 1:**
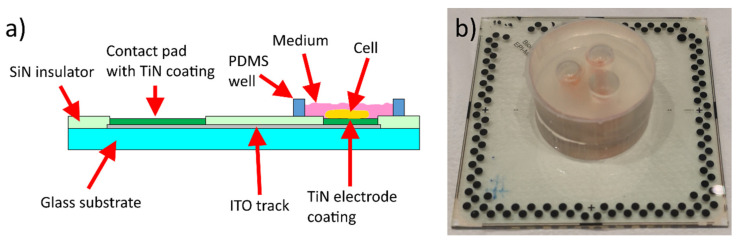
(**a**) Sideview of the ion beam assisted electron beam deposited (IBAD) indium tin oxide (ITO) MEA structure. The image is not to scale. The structure is the same in each batch and version except that the thickness of the ITO track (grey) and/or its deposition method are varied in different batches and the existence or thickness of the TiN coating (green) is varied in different versions. See Table 1 and Table 2 for more details. (**b**) Photograph of an IBAD ITO MEA with a polydimethylsiloxane (PDMS) microfluidic device. The dimensions of the glass substrate are 49 mm × 49 mm.

A total of four batches of MEAs (Table 1) were fabricated and each batch included three versions of MEAs (Table 2). In batch 1 two MEAs of each version were fabricated and in the other batches one MEA of each version. The vertical structure of the MEAs is illustrated in Figure 1a. In version 1, both tracks and electrodes were patterned on an ITO layer. To decrease the impedance and the noise level of the electrodes, the ITO electrodes were coated with a 40 nm TiN layer in version 2 or with a 200 nm TiN layer in version 3. The thin film thicknesses presented in this paper are nominal values measured by the quartz crystal-based thickness controller of the deposition system. Stylus profilometer (Bruker DektakXT) measurements were occasionally performed to check that the thickness was within ~±10% limits. TiN was also applied to the contact pads in each MEA version to increase their thickness and thus make them less prone to be punched by the contact pins. A custom layout included 72 circular measurement electrodes with typical 30 µm diameter and 180–200 µm center-to-center distance. There were also 24 square measurement electrodes with 35 × 50 µm^2^ area and 180 µm center-to-center distance and a large grounding electrode shared between three areas containing an equal amount of both measurement electrode types. The MEAs were compatible with the Multi-Channel Systems’ (MCS, Multi-Channel Systems MCS GmbH) 120-electrode MEA format.

The MEA fabrication process was started by cleaning 49 mm × 49 mm × 1 mm soda lime glass wafers (Gerhard Menzel) with acetone and isopropanol in an ultrasound bath. The ITO layer was deposited on the wafers by the IBAD method, where 95/5 ITO pellets (g-materials) were used as source material and evaporated by e-beam at ~0.24 Å/s deposition rate. During the deposition, the wafers were bombarded with oxygen and argon ions from Saintech ST-55 ion source (Telemark). The gas flow for both gases was 5 sccm (standard cubic centimeters per minute) and the ion current was ~1.1 µA. The fabrication was continued by a lift-off process for titanium alignment marks. After that, electrodes, tracks and contact pads were patterned to the ITO layer by reactive ion etching (RIE) using argon as etching gas and positive photoresist (Futurrex PR1-2000A1) as etching mask. An insulator layer of 500 nm of silicon nitride (SiN) was then deposited at 300 °C by plasma enhanced chemical vapor deposition (PECVD). Positive photoresist was used as the etching mask in etching the openings for electrodes and contact pads by a RIE (Advanced Vacuum Vision 320) process using SF_6_ and O_2_ as the etching gases. The fabrication of the different versions of the MEAs was finalized by a lift-off process of IBAD deposited TiN as described earlier in [23]. In the case of version 1, the electrodes were protected by a drop of photoresist prior to TiN deposition and thus in version 1 only the contact pads have a TiN layer.

In the literature, the track thickness in MEAs is, typically, a couple of hundred nm [23,24,25]. However, in this study, in batches 1 and 2, the IBAD ITO thickness was only 20 nm. The motivation for this was the rarely-reported, but commonly-known increased leakage risk of silicone tunnel structures when crossing over the tracks. Using thinner tracks and thus a flatter MEA surface might help to reduce the risk. For comparison, especially to study the role of the track thickness and conductivity on track impedance, two more batches of MEAs were fabricated. The electrode top layers remained the same in each batch, while the underlying track layer was modified. In batch 3, the IBAD layer thickness was increased to 150 nm and in batch 4, commercial 0.7 mm thick boro-aluminosilicate glass wafers with sputter-deposited ITO (University Wafer Inc.) were used as the starting point. In these MEAs, the ITO thickness was 180 nm and the sheet resistance was as low as 8–10 Ω/sq, according to the manufacturer.

### 2.2. Technical Characterization

Transmission spectra in the visible wavelength range (380–800 nm) of the 20 nm ITO layer as well as the ITO layer + the thin 40 nm TiN layer were measured with JAZ spectrometer (Ocean Optics). Transmission of the thicker 200 nm TiN layer was not measured because of its obvious opaqueness. Both samples were randomly selected from different deposition batches than those used for fabricating the MEAs.

The impedances of all the 72 + 24 measurement electrodes of each MEA were measured at 1 kHz frequency with MEA-IT120 impedance test device (MCS). In the case of batch 1, which was also the batch used in the cell experiments, the impedances were measured before the cell experiments. Dulbecco’s phosphate-buffered saline (DPBS, Merc) was used as the medium in the impedance measurements. The results are presented as a mean ± standard deviation of each functional electrode of the same MEA type and electrode size. Electrodes whose impedance was greater than twice the median impedance or less than half of the median impedance were excluded from the calculations as faulty electrodes. Area-normalized impedance values were calculated by assuming that the impedance is inversely proportional to the electrode area.

**Figure 2:**
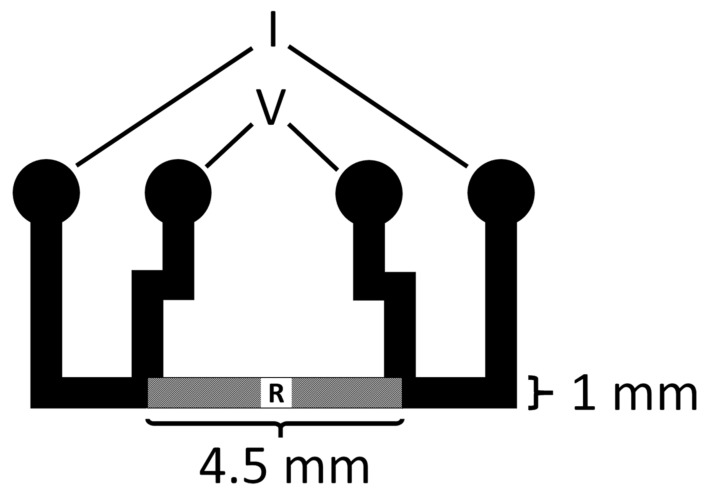
Four-wire test pattern for sheet resistance measurements.

The noise levels of each MEA version of batch 1 were approximated by calculating the root-mean-square values from the 10 min cell recording data on day 10 of the cells on MEA. Conductivities of IBAD ITO thin films were evaluated by measuring sheet resistances from test samples. A simple millimeter-sized pattern for 4-point measurement (Figure 2) was deposited through a mechanical mask on a microscope slide and the measurement was done by a Keithley 2002 digital multimeter.

### 2.3. Cell Experiments

A polydimethylsiloxane (PDMS) microfluidic device with three cell culture medium compartments was bonded onto the MEAs before plating the cells (Figure 1b). A human embryonic stem cell (hESC) line Regea 08/023 for neuronal cell production was used in this study. The Faculty of Medicine and Health Technology has the approval from the Finnish Medicines Agency (FIMEA) to perform research using human embryos (Dnro 1426/32/300/05). The cortical neural differentiation was performed by the recently optimized method [26]. The neuronal cell plating to the devices was performed after 32 days of predifferentiation. This day is considered as day zero on the devices [26]. The cells were plated in a density of 290,000/cm^2^ to each compartment. All medium in the medium reservoirs (200 µl/compartment) was changed a day before MEA recordings and in total four times per week. A control cell culture was grown at the same time on standard plastic plates.

The neuronal activity in the devices was recorded using a MEA2100 system (MCS). The temperature of the MEA headstage was kept at a steady +37 °C using TC02 temperature controller (MCS). The medium compartments were covered with a sterile transparent plastic film to prevent evaporation of medium and contamination during the measurements. The standard 10 min recordings for monitoring the development of the network activity were performed twice a week, up to seven weeks (84 days in vitro, 52 days on MEA). The raw data was obtained at the sampling rate of 25 kHz.

## 3. Results

**Figure 3:**
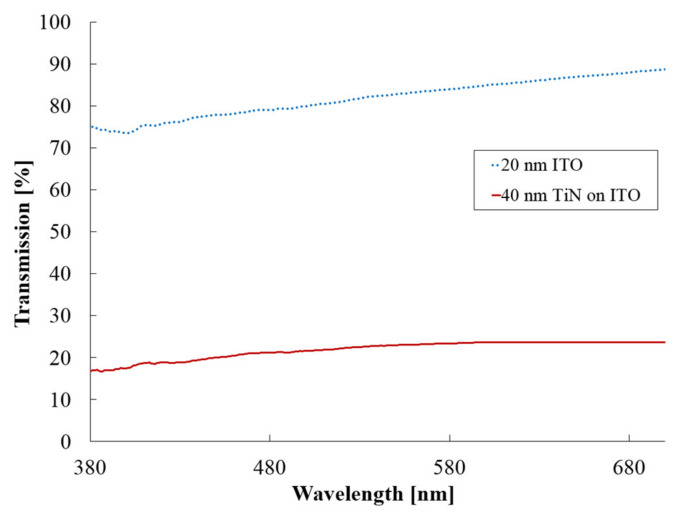
Transmission of IBAD deposited 20 nm ITO and 40 nm TiN films at visible wavelengths.

The transmission spectrum (Figure 3) showed an almost linear increase in transmission from 74% to 89% for the 20 nm IBAD ITO layer within the studied wavelength range. When a 40 nm IBAD TiN layer was added, the transmission dropped to 17%–24%. Importantly, partial transparency was still maintained unlike with the thicker 200 nm IBAD TiN layer, whose transmission was not measured for its obvious opaqueness.

Impedance measurement results as well as noise level and sheet resistance estimates are summarized in Table 3. Briefly, version 1 with bare ITO electrodes clearly showed the highest impedance, from 1200 kΩ to 1950 kΩ depending on the batch. The errors in these MEAs were also relatively high. In version 2, the thin TiN layer resulted in a drop of the impedance to one quarter or even more when compared to the bare ITO electrodes, ranging between 300 kΩ to 370 kΩ. The thick TiN layer in version 3 resulted in a decrease of the impedances to ~200 kΩ. In square electrodes, possessing a larger area, the impedance values were lower as expected, roughly 50% lower compared with the circular Ø30 µm electrodes. Depending on the MEA, 4–13 (5%–18%) of the 72 circular Ø30 µm electrodes were considered faulty and excluded from the results. The fault was usually caused by a scratch, dirt, or failed lift-off type of fabrication damage while no correlation to ITO thickness nor its source were observed.

**Figure 4:**
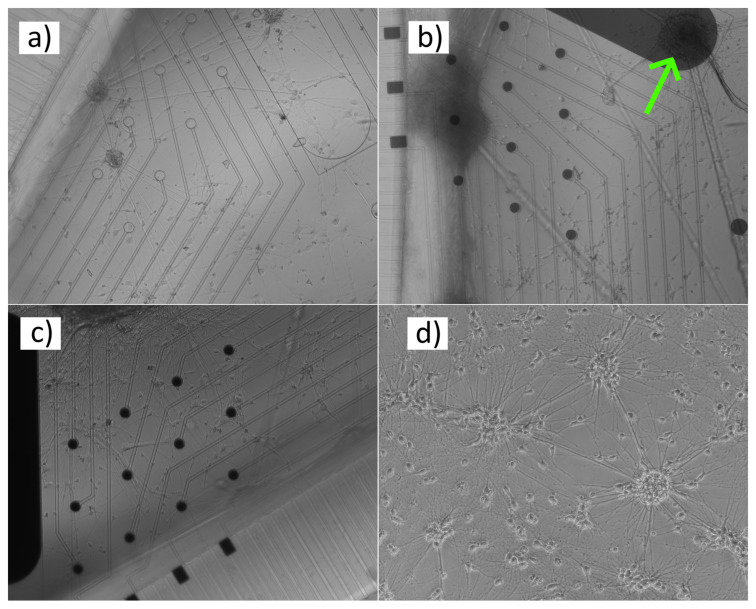
Inverted microscope images of neuronal cell networks growing on different versions of batch 1 MEAs (**a–c**) and a control culture (**d**) grown at the same time on standard plastic plate (29 days after plating). (**a**) The cells are fully visible through the tracks and circular electrodes of MEA version 1. (**b**) In MEA version 2 the visibility through the electrodes drops to partial. However, the cells above the electrodes can still be seen (see especially the larger cluster on the grounding electrode, pointed out by the arrow). (**c**) No visibility through the electrodes in MEA version 3. The control image was taken with a different microscope than the MEA images.

**Figure 5:**
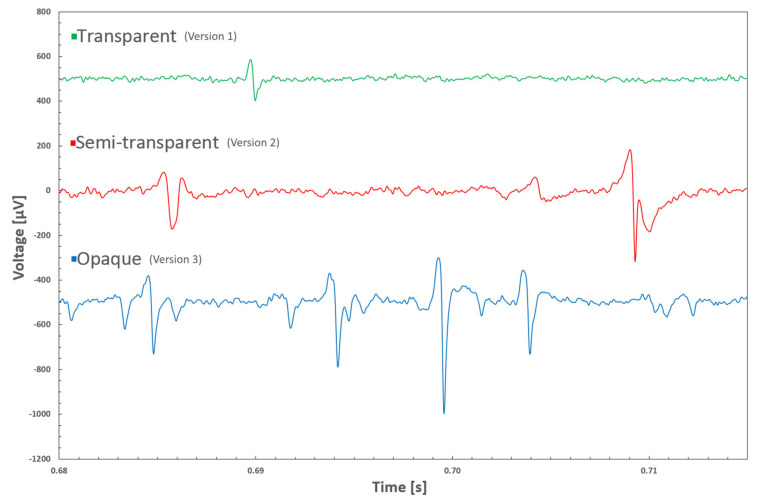
Examples of neuronal cell field potential recordings with different MEA versions from batch 1. Offsets of the curves have been shifted for clarity.

In the cell experiments, no biocompatibility issues were observed and the neurons grew well during the seven weeks of culture time. Microscopic imaging with the inverted microscope was also successful. With the inverted microscope, it was possible to observe the cells through the electrodes in versions 1 and 2 (Figure 4a,b). In version 3, that was not possible. However, due to the transparent tracks, only the cells located directly on top of the electrodes were invisible (Figure 4c). On the control plastic plate (Figure 4d) the cell density after 29 days was a bit higher than on the MEAs, which was expected, as the cells are known to favor plastic vs. SiN/glass MEA surface. The field potentials with all three MEA versions were successfully recorded (Figure 5). In version 1, the signal peaks were clearly lower compared to the other two versions, which provided signal-to-noise ratio comparable to what is typically seen both with commercial or in-house made MEAs with Ti tracks and TiN electrodes [23].

## 4. Discussion

The fabrication of MEAs with ITO or TiN electrodes has traditionally required access to a sputter coater with corresponding targets and preferably the possibility to run a reactive sputtering process with oxygen and nitrogen gases. In this paper, we have shown that ITO electrodes can be fabricated using an e-beam coater equipped with an ion-source, i.e., by the IBAD method. An alternative for sputtering thus exists.

For opaque TiN electrodes, we have shown the usability of the IBAD method in an earlier paper [23]. Similarly, a decrease in impedance while maintaining (partial) transparency by using a thin TiN layer has been shown earlier to be doable by sputtering [15] and atomic layer deposition (ALD) [14]. In this paper, we show for the first time that transparency of electrodes with reasonable impedance can also be achieved with the IBAD method. Even if the main criteria for the method selection is the availability of the deposition systems, IBAD provides some advantages in terms of raw material costs as one can get started with just two crucibles of pellets compared with large and expensive sputtering targets or the usually not-so-cheap ALD precursors. Furthermore, if a TiN layer is applied, as an e-beam deposition-based line-of-sight method IBAD has better lift-off compatibility than more conformal sputtering [27] and ALD [28] deposition methods. The downside of IBAD is its compromised thickness control compared especially with ALD, in which the thin film thickness can be controlled at atomic layer level [28], at least in theory.

The rather large differences in the impedance values between batch 1 and batch 2, despite their equal parameters most likely originated from the unavoidable differences in the IBAD deposition conditions—our deposition system is manually operated and, in addition, it is also used for many other thin film depositions which typically affect the following depositions. Batches 2–4 were fabricated within a short time and their deposition conditions should be more comparable than the ones of batch 1, fabricated one year earlier. However, for the high error in the impedances of version 1 MEAs, i.e., the MEAs without TiN coating and thus with high impedance (1200–1950 kΩ), the most evident explanation is the MEA-IT impedance tester device. We have noticed that it has difficulty in measuring high impedances in a reliable and repeatable manner as it is optimized for 1–2 decades lower impedances.

When the impedances of square electrodes were normalized to correspond to the area of smaller circular electrodes (Table 3), excluding one lucky exception, there was no match, but the normalized values were higher than the circular electrode values. This is, however, to be expected as the idealistic assumption of the impedance being exactly inversely proportional to the area is rarely true. Usually the impedance has not only resistive but also capacitive components. Another factor may be that the calculations were made based on nominal electrode sizes and it is possible that in different MEAs and electrode shapes the error to the real sizes varies slightly.

In many electronic devices, conductivity of the tracks or wires has a great influence on the operation and performance of the device. In MEAs, however, the impedance of the double-layer interface [29] between the electrode surface and the medium/cells is so huge compared with the rest of the system that the tracks have practically no influence on the total impedance. This was also proved in this study as neither the ITO track deposition method nor thickness made any difference to the impedance which would not fit within the electrode-to-electrode or batch-to-batch variations. On the contrary, adding a TiN coating to the electrodes and increasing its thickness clearly decreased the impedance. Even if not visible by the simplified and, unfortunately, common industry-standard method applied in this study, e.g., evaluating the magnitude of the electrode impedance at 1 kHz frequency, one may miss more subtle differences. Thus, most likely with more detailed noise and impedance analysis over a wider frequency range, evidence may arise that the track conductivity does matter when the ultimate signal quality, especially at certain frequency ranges, is considered. This is, however, left for future studies, partly because our IBAD ITO process is not yet able to produce as highly conductive thin films as has been reported earlier for other applications [30]. So, another future topic would be improving the conductivity of IBAD ITO thin films.

## 5. Conclusions

To deposit ITO and TiN for MEA tracks and electrodes, IBAD is a valid alternative to sputtering. Depending on the need, the transmission and the impedance of the ITO, electrodes can be tuned by varying the thickness of the TiN layer. A 40 nm TiN layer on ITO electrodes can be considered as a good compromise, providing sufficient transmission in inverted microscope imaging but also relatively low impedance and thus a good signal-to-noise ratio.

## Figures and Tables

**Table 1:** Details of microelectrode array (MEA) batches.

MEA Batch	1	2	3	4
ITO thickness (nm)	20	20	150	180
ITO deposition method	IBAD	IBAD	IBAD	Sputtering

**Table 2:** Details of MEA versions.

MEA Version	1	2	3
Track material	ITO	ITO	ITO
Electrode material	ITO	TiN on ITO	TiN on ITO
TiN thickness (nm)	-	40	200

**Table 3:** Impedances at 1 kHz and estimates of RMS noise level and sheet resistance. In the normalized column the impedances of 35 × 50 µm^2^ electrodes are normalized to correspond to the area of Ø30 µm electrodes.

Batch/ Version	ITO Deposition Method	ITO Thickness (nm)	TiN Thickness (nm)	Measured Impedance of Ø30 µm Electrodes (kΩ)	Measured Impedance of 35 × 50 µm^2^ Electrodes (kΩ)	Normalized Impedance (kΩ)	RMS Noise (µV)	ITO Sheet Resistance (Ω/□)
1/1	IBAD	20	-	1200 ± 260	820 ± 140	2030	12 ± 1	2.6 × 10^3^
2/1	IBAD	20	-	1950 ± 810	1170 ± 120	2900	Na	9.4 × 10^6^
3/1	IBAD	150	-	1940 ± 290	1180 ± 150	2920	Na	76.4 × 10^3^
4/1	Sputtering	180	-	1420 ± 170	830 ± 30	2050	Na	8–10 *
2/1	IBAD	20	40	320 ± 20	190 ± 10	470	6.1 ± 0.5	2.6 × 10^3^
2/2	IBAD	20	40	370 ± 10	150 ± 10	370	na	9.4 × 10^6^
3/2	IBAD	150	40	350 ± 10	160 ± 3	400	na	76.4 × 10^3^
4/2	Sputtering	180	40	300 ± 10	130 ± 2	320	na	8–10 *
1/3	IBAD	20	200	190 ± 10	150 ± 20	370	5.6 ± 0.5	2.6 × 10^3^
2/3	IBAD	20	200	240 ± 10	120 ± 2	300	na	9.4 × 10^6^
3/3	IBAD	150	200	210 ± 4	110 ± 2	270	na	76.4 × 10^3^
4/3	Sputtering	180	200	190 ± 3	90 ± 1	220	na	8–10 *

* Data from supplier (University Wafer Inc.).
